# Current and promising therapies based on the pathogenesis of Graves’ ophthalmopathy

**DOI:** 10.3389/fphar.2023.1217253

**Published:** 2023-11-16

**Authors:** Xin Zhang, Qixiang Zhao, Bei Li

**Affiliations:** ^1^ Eye School of Chengdu University of Traditional Chinese Medicine, Chengdu, China; ^2^ Department of Ophthalmology, Chengdu Integrated TCM and Western Medicine Hospital/Chengdu First People’s Hospital, Chengdu, China; ^3^ Key Laboratory of Standardization of Chinese Medicine, Ministry of Education, Chengdu University of Traditional Chinese Medicine, Chengdu, China; ^4^ Department of Physiology and Pathophysiology, School of Basic Medical Sciences, State Key Laboratory of Vascular Homeostasis and Remodeling, Peking University, Beijing, China; ^5^ State Key Laboratory of Biotherapy and Cancer Center, West China Hospital, West China Medical School, Sichuan University and Collaborative Innovation Center for Biotherapy, Chengdu, China

**Keywords:** Graves’ ophthalmopathy, treatment, immune therapy, traditional Chinese medicine, intestinal microbiota

## Abstract

Graves’ ophthalmopathy (GO) is a hyperthyroidism-related and immune-mediated disease that poses a significant threat to human health. The pathogenesis of GO primarily involves T cells, B cells, and fibroblasts, suggesting a pivotal role for the thyrotropin-antibody-immunocyte-fibroblast axis. Traditional treatment approaches for Graves’ disease (GD) or GO encompass antithyroid drugs (ATDs), radioactive iodine, and beta-blockers. However, despite decades of treatment, there has been limited improvement in the global incidence of GO. In recent years, promising therapies, including immunotherapy, have emerged as leading contenders, demonstrating substantial benefits in clinical trials by inhibiting the activation of immune cells like Th1 and B cells. Furthermore, the impact of diet, gut microbiota, and metabolites on GO regulation has been recognized, suggesting the potential of non-pharmaceutical interventions. Moreover, as traditional Chinese medicine (TCM) components have been extensively explored and have shown effective results in treating autoimmune diseases, remarkable progress has been achieved in managing GO with TCM. In this review, we elucidate the pathogenesis of GO, summarize current and prospective therapies for GO, and delve into the mechanisms and prospects of TCM in its treatment.

## Introduction

Graves’ ophthalmopathy (GO) is an autoimmune thyroid associated disease characterized by an inflammatory disorder of the orbit and concurrent hyperthyroidism (Bahn, 2010). The lifetime risk of Graves’ disease (GD) is 0.5% for men and 3% for women. Imaging studies have revealed subtle orbital abnormalities in approximately 70% of patients with GD (Khan et al., 2021). Clinical characteristics of GO include the presence of serum anti-thyroid antibodies and thyrotoxicosis, as well as the existence of auto-reactive lymphocytes. The most prevalent clinical features of GO encompass upper eyelid edema, retraction, erythema of the conjunctivae and periorbital tissues, and proptosis. These distinctive attributes serve as diagnostic criteria for the clinical evaluation of GO. Furthermore, it is worth noting that a subset of patients with GO, roughly comprising 3%–5% of cases, experience a more severe form of the disease. This manifestation is characterized by heightened intensity of symptoms such as severe pain, inflammation, and potentially sight-threatening complications such as corneal ulceration or compressive optic neuropathy (Bahn, 2010). The presence of these severe symptoms significantly impacts both the physical and mental wellbeing of affected individuals, underscoring the urgent need for effective interventions and treatments to mitigate their suffering.

The autoimmune response involving antigen-presenting cells (APCs), T cells, and B cells in GO leads to the production of anti-thyroid stimulating hormone receptor (TSHR) autoantibodies (TRAb). These antibodies infiltrate the thyroid and bind to both the insulin-like growth factor 1 (IGF-1) and TSHR present on thyroid epithelial cells and fibroblasts (Douglas et al., 2008; McLachlan and Rapoport, 2014). This binding triggers the activation of thyroid epithelial cells and fibroblasts, resulting in the release of inflammatory cytokines, which further amplifies the inflammatory response (Armengol et al., 2003). In addition, the activated infiltrating T cells recognize the orbital fibroblasts (OFs) trough CD40 ligand interactions. These T cells then secrete cytokines and chemokines such as IFN-γ, TNF-α, IL-1β (Th1), and IL-4 (Th2). These factors contribute to fibroblast differentiation and the expansion of orbital fat by stimulating the release of glycosaminoglycans, such as hyaluronan, from OFs. This excessive accumulation of glycosaminoglycans leads to the swelling of orbital tissues, particularly the extraocular muscles, a hallmark of GO pathology (Bahn, 2015; Smith and Hegedus, 2016).

For several decades, corticosteroids were considered as the primary treatment approach for GO (Wiersinga, 2017). However, the clinical demand for innovative treatment strategies for GD has prompted development of various new therapies, including biologics, small molecule peptides, and immunomodulators. Additionally, a growing body of research has started to explore the therapeutic potential of traditional Chinese medicine (TCM) for GO, owing to its distinctive healing properties and associated mechanisms of action (He et al., 2022). We have compiled a comprehensive overview of current investigations into the utilization of TCM for GO treatment, shedding light on their regulatory mechanisms. Ultimately, we present a forward-looking perspective on GO treatment possibilities, encompassing areas such as gut microbiota.

## Pathogenesis of GO

Researchers classified cells in GO orbital connective tissues into six independent cell types—lymphocyte (mainly T and B cells), APCs, OFs, endothelial cells, adipocytes, and myocytes—using single cell sequencing analysis and multicolor flow cytometry (Fang et al., 2019). Disruption of self-tolerance to the TSHR results in the recognition of TSHR epitopes by APCs and B cells which activate naïve T helper (Th) cells. During above process, medications designed to target APCs, such as tocilizumab (an anti-IL6R agent that focuses on dendritic cells), have been developed with the aim of preventing excessive activation of APCs. Once activated, T cells differentiate into various subsets of Th cells that secrete different cytokines and inflammatory factors, thereby promoting the expansion and intensification of inflammation. Therapies aimed at targeting these inflammatory factors and cytokines serve to halt further inflammation. Examples include adalimumab (targeting TNF-α) and belimumab (targeting BAFF). Moreover, in addition to cytokine targeting, drug interventions also focus on cell–cell interactions. For instance, iscalimab, an anti-CD40 antibody, impedes the activation of B cells by T cells through the CD154-CD40 interaction pathway. Both activated T cells and cytokines play a role in stimulating the production of thyroid-stimulating hormone receptor autoantibodies (TRAb) by plasma cells derived from self-reactive B cells. These TRAb then stimulate orbital fibroblasts (OFs), triggering immune responses within the orbit. Additionally, CD34^+^ OFs, originating from peripheral fibrocytes, contribute to the inflammation process by producing chemokines and releasing a substantial quantity of cytokines, including IL-1β and prostaglandin E2 (PGE2), which further exacerbate the inflammation within the orbital tissues (Fang et al., 2021). Certainly, the pathogenesis of GO is intricate and involves a network of interactions among various cell types. As a result, the effects of many drugs are not limited to a single cell type but have broader impacts. For instance, Resveratrol, an active ingredient from *Reynoutria japonica Houtt*, reduced the number of adipocytes in GO OFs *in vitro* by increasing the expression of the c-Jun NH2-terminal kinase (JNK) and transcriptional regulators phosphor–extracellular signal-regulated kinase (ERK) which are two important pathways in the regulation of metabolic reprogramming. In this section, we summarized the role of T and B cells and fibroblasts in the pathogenesis of GO. In the subsequent part, we will provide a detailed exploration of how drugs targeting GO operate, drawing upon the mechanisms associated with these pathogenic processes.

### T cells

An early study showed that the infiltration of CD3^+^ cells were observed in the orbital tissues of GD patients which provided evidence of T cells infiltrating the inflamed orbit (Fang et al., 2021). While both CD8^+^ and CD4^+^ cells participate in the infiltration of orbital tissues and contribute to immune regulation, the prevailing belief is that CD4^+^ cells play a more pivotal role in the inflammatory process of GO (Zhang et al., 2021). Research has suggested that the type 1 immune response by Th1 may dominate in the early active of GO and the type 2 immune response by Th2 possibly plays an important role in late inactive GO (Aniszewski et al., 2000). The secretion of IFN-γ by Th1 cells has been found to elicit several effects within the context of GO. It induces a shift of fibroblasts to the G0/G1 phase of the cell cycle, leading to changes in their activity. Additionally, IFN-γ upregulates the expression of CD40 on human fibroblasts, thereby influencing immune interactions. Moreover, IFN-γ enhances the synthesis of hyaluronan, particularly through the CD40^−^CD40L signaling pathway, in fibroblasts of GO. This signaling pathway contributes to the accumulation of hyaluronan, which is associated with the swelling of orbital tissues. Furthermore, IFN-γ strengthens the IL-1β-induced synthesis of hyaluronan in OFs of GO by promoting the expression of the hyaluronan synthase-2 gene. This process contributes to the inflammatory response characteristic of GO (Han and Smith, 2006). In addition to its direct effects, the expression of IFN-γ plays a role in the Th1 immunity-mediated inflammatory network in GO. It increases the secretion of chemokines CXCL9, CXCL10, and CXCL11 by both GO OFs and GO OF-differentiated adipocytes. These chemokines further contribute to immune cell recruitment and inflammation within the orbital tissues (Antonelli et al., 2006).

While IL-4 secreted by Th2 may not directly upregulate the expression of CD40 in fibroblasts, it does inhibit the activation of Timp1 promoter by IL-1β, which reduces the expression of TIMP-1 in GO OFs, suggesting its crucial role in GO. IL-4 inhibits the secretion of PGE2 from the OFs of GO while promoting IL-1β-induced synthesis of hyaluronan in fibroblasts by increasing the expression of hyaluronan synthase-2 gene which indicates the opposite mechanism of action (Han and Smith, 2005).

Elevated serum levels of IL-17A, IL-23, and IL-6 have been observed in GO patients, highlighting the significance of the Th17 pathway and IL-23/IL-17 axis in the progression of this condition (Kim et al., 2012a; Fang et al., 2016). In GO, the Th17 cell lineage dominates; in moderate to severe GO, Th17.1 cells independently express retinoic acid receptor-associated orphan receptor-γt (RORγt) and produce IL-17A; and in severe GO, RORγt and T-bet double-positive Th17.1 cells produce IFN-γ (Fang et al., 2020). In addition, a recent study has provided evidence that IL-17A, rather than IFN-γ, stimulates TGF-β-initiated myofibroblast differentiation. This study also suggests that both CD90^+^ and CD90^−^ OFs contribute to the differentiation of Th17 cells through production of PGE2. Importantly, this effect can be mitigated by the administration of indomethacin, a non-steroidal anti-inflammatory drug (Fang et al., 2017).

### B cells

B cells undergo a transformation into antibody-producing plasma cells, a process that requires a secondary signal which is typically acquired through interactions with T cells. As a result of these interactions, plasma cells that originate from activated B cells begin to secrete TRAb against TSHR. Furthermore, the process of autoantibody class switching, encompassing immunoglobulin classes such as IgE, IgM, and IgG, is facilitated by IL-4 secretion from activated T cells, predominantly from Th2 cells (Lehmann et al., 2008; Davies et al., 2020). A recent study has demonstrated that the blocking of CXCL13 or CXCR5 using neutralizing antibodies leads to a reduction in migration of B cells in GO. This observation suggests that, beyond their antibody-secreting function, B-cell migration also plays a pivotal role in the pathogenesis of GO (Wan et al., 2021).

### Fibroblasts

The fibrocytes derived from bone marrow differentiate into CD34^+^ fibroblasts, which in turn can further specialize into either adipocytes or myofibroblasts. These CD34^+^ fibroblasts coexist within the orbital tissue alongside resident CD34^−^ fibroblasts. Stimulation with IL-1β, IL-6, TNF-α, and CD40 ligand-secreted by T cells, B cells, and fibrocyte prompts the activation of CD34^+^ fibroblasts. Additionally, IL-17A has been identified as a factor that promotes TGF-β-induced fibrosis in CD90^+^ OFs and impedes 15-deoxy-Δ12,14-prostaglandin J2-induced adipogenesis in CD90^−^ OFs. In addition, the study highlights that Th17 cells contribute to the secretion of proinflammatory cytokine in both CD90^+^ and CD90^−^ OFs, thus substantiating their role in fostering inflammation (Fang et al., 2017).

## Current treatment

### Antithyroid drugs (ATD)

Thionamide-derived ATD approved for the treatment of patients with Graves’ hyperthyroidism include methimazole (MMI), carbimazole (which is converted to MMI after absorption), and propylthiouracil (Bartalena, 2013). ATD have been widely recommended for patients worldwide as the preferred method of treatment. They work by inhibiting iodination, a process catalyzed by thyroid peroxidase, which in turn downregulates the production of thyroid hormones. Among these derivatives, MMI stands out as a classical and extensively used antithyroid drug (Brix et al., 2020). Compared with MMI, both carbimazole and propylthiouracil have demonstrated lower efficacy in reducing thyroid hormones. Furthermore, these two drugs are available in significantly smaller quantities worldwide. Although ATD, including MMI, have been regarded as the standard therapeutic approach due to their high efficacy, acceptability, low side effects, and extended half-life, they have also exhibited a range of adverse effects (Cooper, 2003). The initial remission rate for first-line treatment with ATD stood at approximately 45.3% (351/774). However, when a second round of ATD was administered to patients who had experienced a relapse after initial treatment, the remission rate decreased further, reaching 29.4%. A lower response rate implies a higher likelihood of relapse, which in turn elevates the patient’s risk of developing goiters (Starling, 2019).

### Glucocorticoids

Glucocorticoids have been established as the primary treatment for managing active disease. Studies have indicated that administration of methylprednisolone effectively suppresses key pathological factors including prostaglandin secretion, production of glycoaminoglycan (GAG), fibroblast activity, and the expression of pro-inflammatory cytokines in the orbital tissue (Zang et al., 2011). Although oral glucocorticoid (GC) has been a longstanding treatment option for GO, recent research indicates that intravenous drug delivery (IVGC) offers enhanced efficacy. Intravenous administration has also been associated with fewer and milder side effects compared to oral administration. These side effects include secondary adrenal insufficiency, cushingoid features, elevated blood pressure, weight gain, hirsutism, muscle pain, depression, and osteoporosis (Macchia et al., 2001; Zang et al., 2011). However, fatal acute hepatotoxicity has been reported in four GO patients treated with IVGC, suggesting that glucocorticoids are not a perfect treatment strategy for GO (Le Moli et al., 2007).

## Immunotherapy targeting cytokines

### BAFF

Belimumab, an anti-B cell activating factor (BAFF) monoclonal antibody (mAb), directly interacts with transitional B cells and thus antagonizes the bioactivity of soluble BAFF (Salvi, 2014). BAFF is indeed a member of the tumor necrosis factor family, which controls the survival and proliferation of B cells. And blocking the interaction between BAFF and its receptor indirectly reducing the survival rate of B cell and reducing the production of TRab (Stohl et al., 2012; Campi et al., 2015).

### TNF-α

The correlation between elevated levels of circulating TNF-α levels and the severity of GO has prompted the exploration of mAb targeting TNF-α. Notably, mAbs such as etanercept, adalimumab, and infliximab have been investigated for their potential in addressing this association (Kumari and Chandra Saha, 2018). Among these, adalimumab has been approved by the Food and Drug Administration (FDA) for the treatment of psoriatic arthritis and inflammatory bowel disease, with notable improvements observed. After 12 weeks therapy of adalimumab, there was reduced inflammation in six of ten GO patients, and increased inflammatory signs in three of the patients (Ayabe et al., 2014). These results highlight the need for further research to comprehensively investigate the role of TNF-α immunosuppressive agents in the context of GO.

## Immunotherapy targeting cell receptors

### CD20

Rituximab was the first biologic therapies applied to the treatment of active GO (Genere and Stan, 2019). Through specifically binding CD20, a molecule exclusively present on B cells, rituximab achieves the depletion of B lymphocytes, leading to a reduction in cytokines and the release of TRAb (Pavanello et al., 2017). This focused inhibitor of CD20 has been identified in case series, indicating a potentially favorable impact on GD and GO (El Fassi et al., 2006; Khanna et al., 2010).

### CD40

Iscalimab (CFZ533), an anti-CD40 mAb, targets the CD40^−^CD154 costimulatory pathway that plays a crucial role in T-cell-dependent immune responses involving activated B cells (Ristov et al., 2018). Unlike rituximab, which depletes B lymphocytes, iscalimab does not induce the depletion of human CD40-expressing B cells. Instead, it disrupts the initial stages of T-cell-dependent antibody responses in non-human primates and inhibits the formation of germinal centers.

### IL-6R

IL-6, along with its soluble receptor, is known to be activated in patients with active GO. This proinflammatory cytokine, which is also excessively expressed in orbital tissues of GO patients, contributes to the inflammatory process. Tocilizumab, a mAb targeting the IL-6 receptor (IL-6R), has been employed as a treatment option. Its administration has shown positive outcomes in alleviating symptoms such as exophthalmos, extraocular muscle mass enlargement, and edema in individuals with GO (Perez-Moreiras et al., 2014; Perricone et al., 2016; Kahaly et al., 2020).

### TSHR

Small molecule TSHR antagonists were reported to specifically target TSHR including Antag-3, S37a, K1-70, and VA-K-14 (Neumann et al., 2010; Neumann et al., 2014). VA-K-14 and S37a have been demonstrated to have the ability to inhibit expression of TSH and TRAb-induced signaling *in vitro* (Latif et al., 2016; Marcinkowski et al., 2019). Antag-3 has shown inhibition of TSH-stimulated cyclic adenosine monophosphate (cAMP) production *in vitro* and a reduction thyroid hormone level in mice treated with thyroid-stimulating mAb M22. By blocking TSHR, K1-70 decreased total T4 and free T4 levels in rats, suggesting its potential as a novel drug to counter TSHR stimulation by TRAb in GD (Furmaniak et al., 2012). An encouraging case report showed that K1-70 monotherapy decreased the thyroid stimulating antibody activity measured in serum as well as improved symptoms (proptosis and inflammation) in a GO patient (Ryder et al., 2021). However, specific immunotherapy for TSHR have broad immunosuppressive effects which may lead to infections.

### IGF-1R

Likewise, activation of the IGF-1R/TSHR protein complex increases the secretion of IL-6 and IL-8 which exacerbates immune responses and inflammation in GO. Teprotumumab, a human monoclonal antibody that blocks IGF-1R, represents a significant breakthrough as the only FDA-approved drug for treating GO. By blocking IGF-1R/TSHR crosstalk, teprotumumab decreases the synthesis of hyaluronate and adipogenesis on the surface of OFs and achieves therapeutic effects for GO (Smith et al., 2017; Antonelli et al., 2020; Krieger et al., 2022).

## Immunotherapy-targeted blocking of immunoglobulin

### FcRn and IgG-1

**FIGURE 1:**
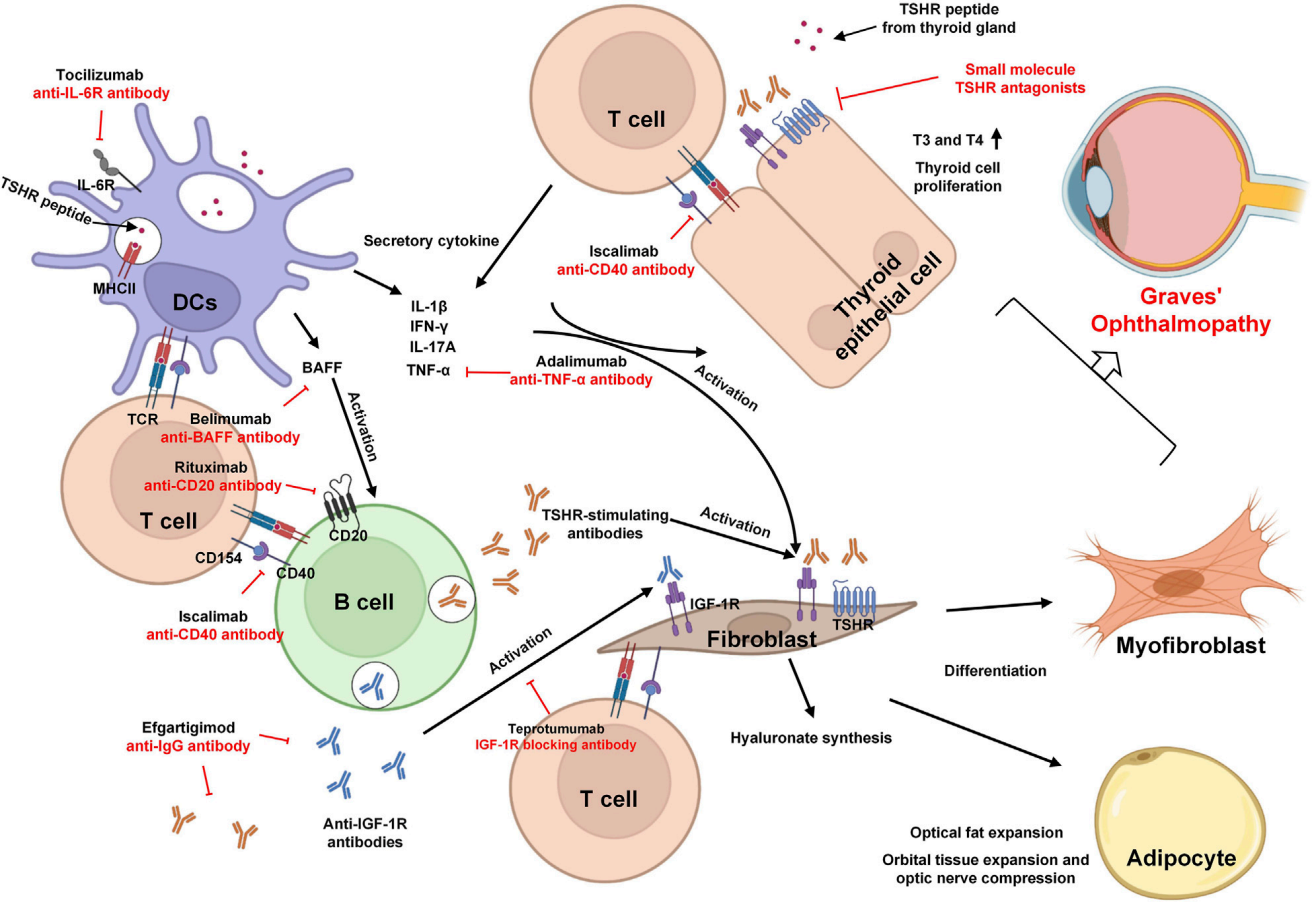
The pathogenesis and immunotherapy strategies of GO.

By blocking the FcRn-IgG-mediated interaction, drugs like rozanolixizumab (an anti-FcRn mAb) and efgartigimod (a humanized IgG-1 derived Fc fragment) hold promise as potential therapeutic options for GO (Kiessling et al., 2017; Smith et al., 2018). Inhibiting FcRn presents an appealing avenue for novel therapy, where accelerated antibody breakdown and reduced levels circulating pathogenic TRAb align with effective treatment for GD (Zuercher et al., 2019). The pathogenesis and immunotherapy strategies of GO are summarized in Figure 1.

## TCM and other treatments

### Polydatin

Polydatin (PD) serves as the primary active ingredient of *Polygonum cuspidatum Sie*, renowned for its anti-inflammatory and neuroprotective properties. In the context of neuropathic pain induced by vincristine (VIN) in rats, PD was observed to curtail the levels of TP53, IL-6, and MAPK1 (Xi et al., 2022). Further investigations employed an *in vivo* model involving adenovirus-induced GO mice expressing the TSHR A-subunit (Ad-TSHR289), as well as an *in vitro* study using hydrogen peroxide (H_2_O_2_)-induced oxidative stress on OFs. In both scenarios, PD exhibited a capacity to mitigate the expansion of orbital muscle adipose tissue and reduce the accumulation of lipid droplets. These effects were attributed to a nuclear factor E2-related factor 2 (NRF2)-mediated response to oxidative stress (Li et al., 2020c).

### Diosgenin (Dio)

Diosgenin, a naturally steroidal saponin found abundantly in various medicinal plants, is notably produced in significant quantities in *Trigonella foenum-graecum L* (He et al., 2012). Administration of Dio for 24 days dose-dependently reduced excessive thyrocyte proliferation and reversed the morphological changes in thyroids by reducing the thyroid size and T4 levels in GD mice while not affecting the abnormal level of TRAb (Cai et al., 2014). In addition, Dio has demonstrated inhibitory effects on the activation and phosphorylation of Rap1-mitogen-activated extracellular signal-regulated kinase (MEK) and PI3K-AKT signaling pathways and promoted cell apoptosis and GD remission. In terms of phosphorylation deactivation in IGF-1R, Dio promoted Nthy-ori three to one cells (normal thyroid cells) apoptosis *in vitro* and relieved GD in rats (Xin et al., 2021).

### Resveratrol

Resveratrol, the active compound derived from *Reynoutria japonica Houtt*, has been shown to have beneficial effects. When combined with PD, it has demonstrated a capacity to decrease the levels of proinflammatory cytokines IL-6, IL-8, and TNF-α in HaCat cells (Ravagnan et al., 2013). Furthermore, resveratrol treatment has been found to effectively reduce the production of reactive oxygen species (ROS), suppress adipogenesis, and reduce the number of adipocytes in GO OFs *in vitro* by increasing the expression of the c-Jun NH_2_-terminal kinase (JNK) and transcriptional regulators phosphor–extracellular signal-regulated kinase (ERK) (Kim et al., 2015).

### Icariin

Icariin, a flavonoid isolated from *Epimedium*, has demonstrated a broad spectrum of effects, particularly its impact on lipid metabolism and reduction, suggesting its important role in the regulation of GO in adipocytes (Wang et al., 2020). Treatment of icariin inhibited the differentiation of preadipocytes into mature adipocytes by suppressing the autophagy which were regulated by the inhibition of AMP-activated protein kinase (AMPK)/mTOR pathway activation *in vitro*. In addition, it reduced lipid droplet accumulation and orbital muscle adipose tissue expansion by inhibiting AMPK/mTOR mediated autophagy in a TSHR-induced GO mouse model (Li et al., 2017).

### Celastrol

Celastrol, a triterpenoid compound derived from TCM sources like Celastrus orbiculatus Thunb, has emerged as a promising candidate for the treatment of diverse inflammatory and autoimmune disorders. Research has illuminated celastrol’s potential in modulating these conditions. A study showed that celastrol reduced the expression of IL-6, IL-8, intercellular adhesion molecule-1 (ICAM-1), and cyclooxygenase (COX)-2, as well as inhibited IL-1β-induced increases in the expression of IL-8, IL-6, COX-2, and ICAM-1. Additionally, the levels of PGE2 (mediated by COX-2) in OFs induced by IL-1β were also suppressed by celastrol (Li et al., 2016).

### Gypenosides

Gypenosides, saponins derived from Gynostemma pentaphyllum, exhibit notable anti-inflammatory properties. In a study involving the glial cell line C6 stimulated by a combination of TNF-α and lipopolysaccharide (LPS), gypenosides significantly attenuated the production of inducible nitric oxide synthase (iNOS), COX-2, IL-6, IL-1β, and TNF-α, underscoring their anti-inflammatory potential (Wang et al., 2017). GO and KEGG (Kyoto encyclopedia of genes and genomes) pathway analysis revealed that gypenosides’ potential curative effect on GO may work via the JAK-STAT pathway and interleukin pathways (Li et al., 2019). Additionally, bioinformatics analyses highlighted the association of gypenosides with fibrosis-related and inflammation-related target genes in GO. This was corroborated by experimental evidence, indicating that gypenosides downregulate inflammatory cytokines (IL-8, IL-6, TNF-α, and CCL2) and fibrotic mediators (HAS2, COL1A2, FN1, and α-SMA) in OFs induced by IL-1β and TGF-β. This effect is achieved by reducing the activation of toll-like receptors (TLRs) 4/NF-κB signaling and TGF-β1/SMAD2/SMAD4 signaling in GO OFs (Li et al., 2020b). A recent study reported that celastrol decreased the oxidative stress level of OFs generated by H_2_O_2_-reduced cell autophagy as well as apoptosis of OFs (Ma et al., 2022). This suggests a multifaceted impact of gypenosides in regulating various processes associated with GO.

### Astragaloside IV

Astragaloside IV treatment significantly downregulates the expression of IL-1β-induced inflammatory cytokine in OFs *in vitro* as well as attenuated GO orbital inflammation, collagen deposition, fat accumulation, and macrophage infiltration *in vivo* (Li et al., 2018).

### Ingredients from Prunella vulgaris

Using bioinformation analysis, research has elucidated that *Prunella vulgaris* holds potential as a treatment against GO. It is believed to promote apoptosis, suppress proliferation, and mitigate inflammation via the PI3K-AKT pathway, thus positioning *P. vulgaris* as a promising candidate for addressing GO (Zhang et al., 2020b). Further analysis unveiled the interaction of key active ingredients in *P. vulgaris*—quercetin, ursolic acid, and rutin—with numerous targets related to GO. These interactions underscore the significant roles of these compounds in the anti-GO context. Quercetin, a flavonoid phytoestrogen, boasts antioxidant and anti-inflammatory properties and has been linked to reduced proliferation in orbital cells (Lisi et al., 2011; Yoon et al., 2013). Ursolic acid and rutin have been demonstrated to promote apoptosis and regulate immune systems in cell and animal models (Manzoni et al., 2019; Satari et al., 2019; Zhang et al., 2020a; Lin et al., 2020). Moreover, our research demonstrated that *P. vulgaris* polysaccharides, a main component of *P. vulgaris* *,* exert their therapeutic effect on the OFs from GO patients by inhibiting the proliferation and promoting the apoptosis of OFs (Li et al., 2020a).

### Triptolide

Triptolide, a diterpenoid tricyclic oxide composition extracted and purified from the roots of *Tripterygium wilfordii* which has been reported to induce T cell apoptosis, inhibits T cell proliferation, reduces IL-2 synthesis, and inhibits the expression of NF-κB in T cells (Li et al., 2002; Qiu and Kao, 2003). Triptolide relieves the clinical manifestations of diplopia, exophthalmos, and periorbital swelling caused by accumulation of adipose tissue and inflammatory cell infiltration in the orbital and muscle connective tissue. The abnormal expression of human leukocyte antigen (HLA)-DR in fibroblasts is associated with the pathogenesis of GO (Bahn, 2020). An *in vitro* experiment showed that triptolide inhibited IFN-induced activation of retro-ocular fibroblasts (RFs) derived from patients with GO including dose-dependently downregulating the percentage of HLA-DR, ICAM-1, and CD40 positive cells on RFs (Yan and Wang, 2006).

### Bupleurum saponins

Bupleurum saponins, the active component of *Bupleurum falcatum L*, exert strong antioxidant effects which improve hyperthyroidism and related organ damage induced by Levothyroxine (LT4) (Kim et al., 2012b; He et al., 2022). However, there is no direct evidence to prove the efficacy of Bupleurum saponins in the treatment of GO.

### Pingmu Decoction

Pingmu Decoction has been used in the treatment of inactive GO as a TCM for over a decade, exhibiting favorable clinical outcomes. By diminishing the viability of orbital preadipocytes and triggering apoptosis in mature adipocytes via the Fas/Fas L signaling pathway, Pingmu Decoction effectively curbs lipid accumulation and reduces the expression of key regulators like PPARγ and C/EBPα. This outcome implies that Pingmu Decoction might hold therapeutic promise for GO by mitigating orbital adipocyte accumulation (Zhang et al., 2017). Additional research substantiates Pingmu Decoction’s efficacy in mitigating GO progression. This involves the attenuation of preadipocyte proliferation and an increase in adipocyte apoptosis in orbital adipose tissue derived from GO patients (Li et al., 2012).

### Berberine

Berberine, a natural alkaloid with the chemical formula C_20_H_18_NO_4_, originates from *Rhizoma coptidis*, a traditional Chinese plant. Treatment of berberine dose-dependently decreased intracellular lipid accumulation by downregulating adipogenic markers in GO OFs. Additionally, berberine attenuated IL-1β-induced expression of proinflammatory molecules in OFs from both GO and control patients by blocking NF-κB signaling (Diao et al., 2022).

### Neferine

Neferine, derived from the traditional Chinese medicinal plant *Nelumbo nucifera*, has garnered attention for its potential therapeutic applications. It has been shown to induce autophagy by inhibiting PI3K/AKT signaling and triggering the generation of ROS (Poornima et al., 2013). It effectively curtails IL-13-induced inflammation, ROS production, fibrosis, and adipogenic differentiation in OFs derived from GO patients. Notably, Neferine’s anti-inflammatory, antioxidant, and anti-lipogenic effects are accompanied by an upregulation of Nrf2, a pivotal transcription factor that safeguards cells against oxidative stress-induced damage (Li et al., 2021). The TCM and other treatments are summarized in the table (Table 1).

**TABLE 1:** TCM and other treatments of Graves’ ophthalmopathy.

Medicine	Extract from	Effect and mechanism
Polydatin	*Polygonum cuspidatum Sieb*	Relieved orbital muscle adipose tissue expansion and reduced lipid droplet accumulation through NRF2-mediated oxidative stress response and downregulation of IL-6 Li et al. (2020c), Xi et al. (2022)
Diosgenin	*Trigonella foenum-graecum L*	Reduced thyrocyte proliferation, thyroid size, and T4 levels in GD mice. Inhibited the PI3K-AKT and Rap1-MEK signaling pathways and promoted thyroid cell apoptosis He et al. (2012), Cai et al. (2014), Xin et al. (2021)
Resveratrol	*Reynoutria japonica Houtt*	Reduced proinflammatory cytokine IL-6, IL-8, and TNF-α in HaCat cells. Reduced ROS production, suppressed adipogenesis, and reduced the number of adipocytes in GO orbital fibroblasts *in vitro* by increasing the expression of the p-JNK and transcriptional regulators p-ERK Ravagnan et al. (2013), Kim et al. (2015)
Icariin	*Epimedium*	Inhibited the differentiation of preadipocytes into mature adipocytes, lipid droplet accumulation, and orbital muscle adipose tissue expansion by inhibiting AMPK/mTOR-mediated autophagy in GO mouse models Li et al. (2017), Wang et al. (2020)
Celastrol	*Celastrus orbiculatus Thunb*	Reduced the IL-1β-induced expression of IL-8, IL-6, ICAM-1, and COX-2 in OFs from patients with GO Li et al. (2016)
Gypenosides	Gynostemma pentaphyllum	Downregulated IL-8, L-6, TNF-α, CCL, HAS2, COL1A2, FN1, and α-SMA in OFs induced by IL-1β and TGF-β through reducing the activation of TLR-4/NF-κB signaling and TGF-β1/SMAD2/SMAD4 signaling in GO OFs Wang et al. (2017), Li et al. (2019), Li et al. (2020b), Ma et al. (2022)
Astragaloside IV	*Astragalus memeranaceus*	Downregulated inflammatory cytokines, and attenuated collagen deposition, fat accumulation, and macrophage infiltration of GO OFs Li et al. (2018)
Ursolic acid, rutin, and *Prunella vulgaris* polysaccharides	*Prunella vulgaris*	Ursolic acid and rutin promoted apoptosis and regulated immune systems in cell and animal models Zhang et al. (2020b). *Prunella vulgaris* polysaccharides exerted its therapeutic effect on the OFs from GO patients by inhibiting the proliferation and promoting the apoptosis of OFs Li et al. (2020a)
Triptolide	*Tripterygium wilfordii*	Induced T cell apoptosis, inhibited T cell proliferation, reduced IL-2 synthesis, and inhibited the expression of NF-κB in T cells. Relieved the clinical symptoms of GO patients as well as downregulated the percentage of HLA-DR, ICAM-1, and CD40 positive cells in RFs Li et al. (2002), Qiu and Kao (2003), Yan and Wang (2006)
Bupleurum saponins	*Bupleurum falcatum L*	Improved hyperthyroidism and related organ damage induced by LT4 Kim et al. (2012b), He et al. (2022)
Pingmu Decoction	Pingmu Decoction	Pingmu Decoction alleviated GO progression, reduced lipid accumulation, and downregulated the expression of PPARγ and C/EBP α via the Fas/Fas L signaling pathway Li et al. (2012), Zhang et al. (2017)
Berberine	*Rhizoma coptidis*	Decreased intracellular lipid accumulation in GO OFs by blocking NF-κB signaling Diao et al. (2022)
Neferine	*Nelumbo nucifera*	Inhibited IL-13-induced inflammation, ROS production, fibrosis, and adipogenic differentiation in GO OFs. The anti-inflammatory, antioxidant, and anti-lipogenic effects of Neferine were accompanied by an upregulation of Nrf2 Poornima et al. (2013), Li et al. (2021), Wang et al. (2022a)

## Prospective treatment

### Disulfiram

Disulfiram, originally approved as an aldehyde dehydrogenase (ALDH) inhibitor by the FDA for alcohol abuse treatment back in 1951 (Lu et al., 2021), has more recently been investigated for its potential therapeutic applications in GO. Recent research showed that disulfiram dose-dependently suppressed lipid accumulation during adipogenesis in OFs of GO by decreasing the expression of key adipogenic transcription factors, including perilipin-1 (PLIN1), FABP4, PPARγ, and c/EBPα (CEBPA). In addition, it suppressed inflammatory molecule expression induced by IL-1β and showed antifibrotic effects in GO OFs (Wang et al., 2022a). Furthermore, disulfiram dose-dependently inhibited contraction, migration, proliferation, and fibrosis in perimysial orbital fibroblasts (pOFs) collected from eight patients with GO (Wang et al., 2022b).

### Intestinal microbiota

In the past few decades, clinical and animal studies have found a strong link between gut microbes and autoimmune diseases (Honda and Littman, 2016), including autoimmune arthritis (Wu et al., 2010), ulcerative colitis (Zhao et al., 2022), and psoriasis (Zhao et al., 2023). By regulating immune cells and affecting the intestinal barrier, intestinal microbes may play a critical role in the development of GO which is an autoimmune thyroid disease.

Clinical study has demonstrated that, compared with healthy control patients, *Prevotella* and *Veillonella* were increased while *Lactobacillus* was decreased in GD patients. These intestinal microbes have also been found to act as marker bacteria in other autoimmune diseases or to be involved in regulating diseases. *Prevotella* has been reported to be associated with rheumatoid arthritis (RA), and specific antigens of *Prevotella* can shape or promote immune responses in RA joints (Pianta et al., 2017; Pianta et al., 2021). In addition, administration of gentamicin decreased the abundance of *Prevotella* and relieved the pathogenesis of psoriasis-like phenotype in K14-VEGF-induced psoriatic mice (Zhao et al., 2023). At present, there are few systematic studies on the effects of intestinal microbes on GO. Even so, *Prevotella copri* was reported to increase significantly in patients with GO, while the abundance of *Parabacteroides distasonis* exhibited an opposite correlation with TRAb, suggesting a potential protection effect of *P. distasonis* against GO (Shi et al., 2019). The protective effect of *P. distasonis* has also been reported to be associated with psoriasis and multiple sclerosis (Cekanaviciute et al., 2017; Zhao et al., 2023). Vancomycin significantly decreased intestinal microbiota as well as reduced the severity and incidence of both GO and GD. Researchers showed that the reduced orbital pathological symptom was positively correlated with *Akkermansia* spp. Additionally, mice transplanted with fecal microbiota from GO patientsinitially inherited their donors’ microbiota, and the induced GD exacerbated, as did the orbital brown adipose tissue volume increase in TSHR mice (Moshkelgosha et al., 2021). As the second genome of the human body, the gut microbiome is composed of many species, which has great potential for exploitation (Zhu et al., 2010). *Akkermansia muciniphila* reduced body weight and the levels of blood markers associated with liver dysfunction in obese humans while *Lactococcus lactis* was transplanted to patients for the treatment of vitamin K deficiency (Depommier et al., 2019; Liu et al., 2019). Hence, the administration of gut microbes has valuable clinical applications for the treatment of several diseases, including GO. In conclusion, more studies are needed to explore the mechanism of gut microbiota regulation on GO, and to treat GO by targeting gut microbiota and supplementing probiotics.

## Conclusion

In the past decades, based on the gradual in-depth understanding of the pathogenesis of GO, the treatment of GO has gradually increased and changed. Glucocorticoids and anti-TSH drugs have made great contribution to the treatment of GO for quite a long time by reducing the TSH level, decreasing orbital inflammation, reducing orbital adipocyte expansion, and alleviating the progression of GO. The low remission rate of secondary drug use and large side effects of drugs have gradually become a drawback of the above-mentioned drug treatment for GO. As an autoimmune disease, GO is expected to be treated with the regulation of immune cells and cytokines which are the potential targets of GO. Tumor therapy with a PD-1 inhibitor caused adverse effects similar to the symptoms of GO and exogenous PD-L1 reduced orbital inflammation of fibroblasts by inhibiting T cell activity (Sagiv et al., 2019; Liu et al., 2022). Using single-cell RNA sequencing, Wang et al. revealed the novel GO-specific cell type CD4^+^ cytotoxic T lymphocytes (CTLs) which are characterized by chemotactic and inflammatory features (Wang et al., 2021). Granzyme B and IFN-γ secreted CTLs may migrate from the circulation to orbits and trigger orbital inflammation and tissue remodeling. Zhang et al. demonstrated that rapamycin, a mTOR inhibitor, ameliorated orbitopathy and hyperthyroidism by decreasing CD4^+^ CTLs’ accumulation and suppressing their inflammatory in GO mice. In addition, by targeting mTORC1 and decreasing the frequency of CD4^+^ CTLs, rapamycin ameliorated diplopia and orbital inflammation in patients with intractable GO (Zhang et al., 2023). These studies encourage researchers to shift the focus from helper T cells to CTLs.

There are a wide range of proven immunological agents or those under investigation for the treatment of GO, and they have favorable biosafety profiles. However, antibody-based drugs account for a large proportion of immunological agents which tend to be expensive. OFs from active GO displayed hypermethylation of genes that linked to inflammation and hypomethylated genes that linked to adipogenesis and autoimmunity, suggesting the important role of DNA methylation in the progression of GO (Virakul et al., 2020). Additionally, Dottore et al. reported that incubation with anti-TSHR antibodies significantly increased global DNA methylation which is related with cell proliferation in fibroblasts (Rotondo Dottore et al., 2023). Although there has been no clinical and in-depth basic research, DNA methylation-based gene therapy still has great prospects for the clinical treatment of GO.

Oral doxycycline, a broad-spectrum antibiotic, resulted in greater improvement of GO related symptoms after 12 weeks, indicating that gut microbes are closely related to the incidence of GO in clinical patients (Pan et al., 2022). Biscarini et al. reported that, compared with healthy controls, *Actinobacteria* were significantly increased while *Bacteroidetes* significantly decreased in GD/GO patients. *Bacteroides* showed the positive and negative correlations with TSH and free thyroxine. Importantly, the presence of *Clostridiales* correlated with the persistence of TRAb which is predictive of relapse, suggesting that targeting *Clostridiales* may be a means of radical treatment of recurrent GO (Biscarini et al., 2023). Studies in mice have also partially confirmed this result (including increased *Bacteroides* and decreased *Actinobacteria*) (Li et al., 2023). However, at present, the studies on GO and intestinal microbiota are relatively shallow, and the regulatory mechanism of GO should be elaborated from the perspective of multiomics and single-bacteria studies in the future.

What is more, some drugs that are not primarily targeted at GO have also shown promising effects in the treatment of GO. AMPK activity showed a reduction in the orbital tissue of GO patients, and treatment of metformin, an AMPK activator, inhibited fibrosis and the expression of inflammatory molecules in OFs of GO via an AMPK/mTOR signaling pathway (Xu et al., 2022). Based on its potential anti-inflammatory properties, reseachers found that dihydroartemisinin (DHA), a sesquiterpene lactone which is widely used for the treatment of malaria and fever that is extracted from a traditional Chinese herb, *Artemisia annua L*., significantly alleviates pathogenic manifestations in OFs of GO by inhibiting proliferation, fibrosis- and inflammation-related gene expression, and TGF-β1-induced inflammation in OFs via suppression of the ERK and STAT3 signaling pathways (Yang et al., 2022).

Generally, in this review, we introduced the pathogenesis of GO from the perspective of immunity, summarized the current treatment methods of GO, focused on the induction of immunotherapy and TCM treatment of GO, and discussed their relationship with GO and the prospect of treatment of GO from the perspective of intestinal microorganisms.
